# Structural Fetal Anomalies Identified During First-Trimester Screening: A Single-Centre Experience in Romania

**DOI:** 10.3390/medicina62101824

**Published:** 2026-09-22

**Authors:** Maria Cezara Mureșan, Marius Bogdan Mureșan, Dan Dumitrașcu Biriș, Ioan Cosmin Cîtu

**Affiliations:** 1 Department of Obstetrics and Gynecology, Faculty of Medicine, Victor Babeș University of Medicine and Pharmacy, 300041 Timișoara, Romania; citu.ioan@umft.ro; 2 Maternal-Fetal Medicine Center, Strada Bobâlna 47, 300331 Timișoara, Romania; medicinafetala@gmail.com (M.B.M.); andreidandumi@yahoo.com (D.D.B.)

**Keywords:** first-trimester ultrasound, nuchal translucency, congenital anomalies, congenital heart defects, exomphalos, prenatal diagnosis, pregnancy outcome, Romania

## Abstract

*Background and Objectives*: The 11–13^+6^-week scan has evolved from a nuchal translucency (NT) measurement into an early anatomical survey, and the anomalies it identifies increasingly shape prenatal counselling and management. Data from Central and Eastern European screening populations are scarce. *Materials and Methods*: Retrospective cohort of all pregnancies undergoing first-trimester combined screening in a single fetal medicine centre in Romania between August 2009 and April 2026, with a crown–rump length of 45–84 mm. The centre provides routine screening but also receives women referred after an abnormal finding elsewhere; the cohort is therefore described as a screening population with referral enrichment. Structural anomalies recorded prospectively in the ultrasound database at the first-trimester scan were classified by organ system and by first-trimester detectability category. NT, combined risk, karyotype and pregnancy outcome were extracted from the same database. Associations were quantified with odds ratios (OR) from logistic regression with cluster-robust standard errors by woman. *Results*: 11,229 pregnancies (10,015 women; 408 multiple pregnancies) were included. A structural anomaly was identified at 11–13^+6^ weeks in 197 (17.5 per 1000; 95% CI 15.3–20.1); 16.1 per 1000 (13.8–18.7) after exclusion of the years 2009–2012 and 16.9 per 1000 (14.6–19.5) in singleton pregnancies. A further 44 pregnancies had isolated cystic hygroma/hydrops or soft markers only. Cardiac defects (81; 7.2 per 1000) and abdominal-wall defects (74; 6.6 per 1000, of which 67 exomphalos) were present in 145 pregnancies (74%), followed by central nervous system (27), facial (23), skeletal (23), thoracic (10) and urogenital (10) anomalies; 37 pregnancies (19%) had anomalies in two or more systems. NT ≥ 3.5 mm was present in 59/197 (30%) anomalous versus 203/11,032 (1.8%) non-anomalous pregnancies (OR 22.8, 95% CI 16.3–31.9), and in 51% of cardiac defects. Among 37 karyotyped fetuses, 15 (41%) had a chromosomal abnormality. Pregnancy outcome was known for 87 anomalous pregnancies: 46 terminations, 33 live births and 8 fetal or neonatal deaths; 17 of 30 exomphalos cases with known outcome were live-born. *Conclusions*: In a screening population with referral enrichment, about 1 in 57 pregnancies had a structural anomaly identified at the 11–13^+6^-week scan, dominated by cardiac and abdominal-wall defects; the figure describes prenatally identified anomalies in this setting and is not a population-based prevalence. One third of these fetuses had increased NT and a high proportion of those tested had an abnormal karyotype, supporting the integration of systematic anatomical assessment and genetic evaluation into first-trimester screening.

## 1. Introduction

The 11–13^+6^-week ultrasound examination was introduced as a tool for the measurement of nuchal translucency (NT) in screening for trisomy 21 [1]. Improvements in transducer technology and the systematic use of a standardised anatomical protocol have progressively transformed it into an early anomaly scan [2,3]. Large series from the Fetal Medicine Foundation have shown that a substantial proportion of major non-chromosomal anomalies can be diagnosed at this gestation, and that detectability is strongly anomaly-specific: acrania, alobar holoprosencephaly, exomphalos, gastroschisis, megacystis and body-stalk anomaly are essentially always detectable, whereas many cerebral, renal and gastrointestinal defects are not diagnosed before the second trimester [4,5]. Systematic reviews report a pooled first-trimester detection rate of about 46% for all structural anomalies and 50–55% for major cardiac defects, with wide variation between settings [6,7], and a recent nationwide study of more than one million pregnancies in England found that one third of major anomalies were identified before 16 weeks, with higher rates where a detailed first-trimester protocol was in use [8].

Most published cohorts originate from Western European or Asian tertiary networks with near-complete postnatal ascertainment. Data from Central and Eastern Europe are limited to a few centres [9,10], and there is little information on the spectrum of first-trimester diagnoses, their association with NT and aneuploidy, and their outcome in a screening population followed within the same institution. In Romania, first-trimester combined screening is largely delivered by fetal medicine centres outside the public hospital system, while births and postnatal diagnoses are dispersed across maternity units, so that the experience of these centres is rarely reported.

The aims of this study were (i) to describe the prevalence and spectrum of structural anomalies identified at the 11–13^+6^-week scan in a screening cohort with referral enrichment undergoing combined screening in a single centre between 2009 and 2026, (ii) to examine the association of these anomalies with NT thickness, combined-screening risk and karyotype, and (iii) to report pregnancy outcome where available.

## 2. Materials and Methods

### 2.1. Study Design and Population

This was a retrospective cohort study of all pregnancies that underwent a first-trimester combined-screening examination at the Maternal-Fetal Medicine Center, Timișoara, Romania, between August 2009 and April 2026 (16 years and 9 months; complete calendar years 2011–2025 are used for annual analyses, see Section 2.4). The study is reported in accordance with the STROBE recommendations for observational studies. The centre provides routine first-trimester combined screening to self-referred and physician-referred women and also functions as a regional referral centre for suspected fetal abnormalities. Consequently, a proportion of women attended after an abnormal finding at an outside examination. The indication for referral was not recorded as a structured database field, and referral for a suspected abnormality therefore cannot be identified reliably at the individual-pregnancy level. The cohort is consequently described throughout as a screening population with referral enrichment. All examinations were performed by or under the supervision of fetal medicine specialists holding the Fetal Medicine Foundation certificate of competence in the 11–13-week scan, according to the FMF protocol for NT measurement and risk assessment; the anatomical survey followed the ISUOG recommendations for the 11–13^+6^-week scan current at the time of the examination [2,3], which served as the reference standard for the centre without a separate written local protocol. The structured anatomy checklist of the reporting database was unchanged over the study period. The 2023 update of the ISUOG guideline [3] extended the recommended survey, in particular the cardiac views and the posterior brain, so that the scope of the anatomical survey performed in the centre may have broadened gradually over the period, alongside improvements in equipment and operator experience. Data were recorded prospectively at the time of the examination in the ultrasound reporting database (Astraia, Astraia Software GmbH, Munich, Germany), including biometry, NT, biochemistry, combined risks, the structured checklist of fetal anatomy with anomaly-specific fields, invasive testing results and pregnancy outcome. The analysis used a pseudonymised extract in which patient identifiers were replaced by a one-way hash; no identifiable data left the centre.

The first-trimester cohort comprised all pregnancies with at least one examination at a crown–rump length (CRL) of 45–84 mm or, when CRL was not recorded, at a gestational age of 11^+0^ to 13^+6^ weeks. Where a woman had several pregnancies during the study period, each pregnancy was analysed separately; pregnancies were reconstructed from the sequence of examinations by grouping visits whose estimated date of the last menstrual period fell within 100 days of each other. Multiple pregnancies were identified from the fetus-number and chorionicity fields of the database and were retained in the denominator; anomaly fields refer to the pregnancy (any fetus). Anomalies and outcomes among multiple pregnancies are described in Section 3.2.

### 2.2. Definition and Classification of Anomalies

A structural anomaly at the first-trimester scan was defined as any positive entry in the anomaly-specific fields of the anatomy checklist (skull, brain, face, spine, thorax, heart, abdominal wall, gastrointestinal tract, urogenital tract, limbs) or in the corresponding free-text fields at any examination within the CRL window. Findings were grouped by organ system and into the categories of first-trimester detectability described by Syngelaki et al. [5]: category A, anomalies always detectable at 11–13 weeks (acrania/exencephaly/anencephaly, alobar holoprosencephaly, exomphalos, gastroschisis, megacystis, body-stalk anomaly); category B, anomalies sometimes detectable (encephalocele, spina bifida, facial cleft, micrognathia, diaphragmatic hernia, major cardiac defects, limb defects and others); and category C, anomalies usually not detectable before the second trimester (in this cohort, renal anomalies other than megacystis). Isolated nuchal oedema, absent or hypoplastic nasal bone, aberrant right subclavian artery, absent ductus venosus and pericardial effusion were regarded as markers rather than structural anomalies and were analysed separately; cystic hygroma and generalised oedema/hydrops without an accompanying structural defect were also reported separately. A pregnancy was counted once per organ system and once overall irrespective of the number of anomalies; in the table listing individual entities, a pregnancy with two entities in the same system appears in two rows, so that entity counts within a system may exceed the number of pregnancies with an anomaly in that system. Pregnancies were further classified as having a single recorded entity, two or more entities within one organ system, or anomalies in two or more organ systems. Detectability categories were assigned per entity, so that a pregnancy with a category A and a category B anomaly is counted in both categories. Findings were analysed as recorded at the first-trimester examination; no retrospective re-classification was performed and findings that may have resolved or been revised at later examinations were not excluded, so that the figures represent what was reported to the woman at the time of screening.

### 2.3. Covariates and Outcome

NT (maximum value across first-trimester examinations), CRL, maternal age at the examination, the adjusted combined risk for trisomy 21, the results of chorionic villus sampling or amniocentesis (karyotype, QF-PCR or chromosomal microarray) recorded in the database, and pregnancy outcome were extracted. Outcome was classified as live birth, termination of pregnancy (TOP), fetal loss (miscarriage or intrauterine death) or neonatal death, and was completed from the second-trimester examination records of the same database where the first-trimester record was incomplete. Second-trimester attendance was defined as at least one examination in the centre at 18–24 weeks in the same pregnancy. Invasive testing and deliveries that took place in other institutions were captured only when the result was reported back to the centre and entered in the database.

### 2.4. Statistical Analysis

Prevalence is expressed per 1000 pregnancies with Wilson 95% confidence intervals (CI); proportions within subgroups are given with Wilson 95% CI. Continuous variables are summarised as median and interquartile range (IQR). Groups were compared with the Mann–Whitney U test for continuous variables and Fisher’s exact or chi-squared test for categorical variables. The association between increased NT (≥3.5 mm) or a high combined risk (≥1:100) and the presence of a structural anomaly was quantified as an odds ratio (OR) from logistic regression and as a prevalence ratio (PR) from a Poisson model with robust variance, both with cluster-robust standard errors by woman to account for women contributing more than one pregnancy. Four sensitivity analyses were pre-specified for the revision: (i) one pregnancy per woman (the first in the study period); (ii) exclusion of the years 2009–2012, in which referral enrichment was most apparent; and (iii) restriction to the complete calendar years 2011–2025; and (iv) restriction to singleton pregnancies. The exclusion of 2009–2012 is a temporal sensitivity analysis and does not constitute a referral-adjusted estimate. Anomalous pregnancies with known and unknown outcome were compared on baseline characteristics to assess the representativeness of the outcome-known subgroup. Annual analyses use the complete calendar years 2011–2025; the low-volume start-up period (August 2009–December 2010) and the partial year January–April 2026 are reported in Table S1. Analyses were carried out in Python 3.11 (pandas 3.0, statsmodels 0.14, SciPy 1.13). A two-sided *p* < 0.05 was considered statistically significant.

## 3. Results

### 3.1. Study Population

A total of 11,800 first-trimester examinations in 11,229 pregnancies of 10,015 women fulfilled the inclusion criteria; 128 pregnancies were excluded because all examinations were outside the CRL window. Maternal age was median 31.3 years (IQR 28.1–34.9), with 2767 women (24.6%) aged 35 years or more. CRL was median 66.0 mm (IQR 60.5–72.3) and NT median 1.9 mm (IQR 1.6–2.2); NT was ≥3.5 mm in 262 pregnancies (2.3%). The adjusted combined risk for trisomy 21 was available in 11,078 pregnancies and was ≥1:100 in 339 (3.1%). There were 408 multiple pregnancies (3.6%; 357 twin, 50 triplet and 1 quadruplet) and 10,821 singleton pregnancies. Annual volume increased from 447 pregnancies in 2011 to 949 in 2025; the start-up period August 2009–December 2010 contributed 136 pregnancies and January–April 2026 a further 296 (Table S1). Of the 10,015 women, 1159 contributed more than one pregnancy.

A second-trimester examination at 18–24 weeks in the same centre was documented for 8285 pregnancies (73.8%). Pregnancy outcome was known for 3105 pregnancies (27.7%): 2892 live births, 130 terminations, 79 fetal losses, 3 neonatal deaths and 1 ongoing pregnancy. Outcome ascertainment was highest for pregnancies screened in 2010–2017 (33–69% per year), fell to 21% in 2018 and ranged between 9% and 20% thereafter (Table S1).

### 3.2. Prevalence and Spectrum of First-Trimester Structural Anomalies

A structural anomaly was recorded at the 11–13^+6^-week scan in 197 pregnancies, a prevalence of 17.5 per 1000 (95% CI 15.3–20.1), or approximately 1 in 57 pregnancies. An additional 6 pregnancies had cystic hygroma or hydrops without a structural defect and 38 had soft markers only, giving 241 pregnancies (21.5 per 1000) with any abnormal anatomical finding. Among the 197 pregnancies with a structural anomaly, 150 (76%) had a single recorded entity, 10 (5%) had two or more entities within one organ system and 37 (19%) had anomalies in two or more organ systems. By detectability category, 69 pregnancies had category A anomalies only, 104 category B only, 2 category C only and 22 both a category A and a category B anomaly; thus 91 pregnancies (8.1 per 1000) had at least one category A anomaly and 126 (11.2 per 1000) at least one category B anomaly. A structural anomaly was identified in 14 of the 408 multiple pregnancies (34.3 per 1000; 95% CI 20.5–56.8)—exomphalos in 8 (in 2 combined with central nervous system defects), alobar holoprosencephaly in 2, hypoplastic left heart, atrioventricular septal defect, another cardiac defect and megacystis in 1 each—and in 183 of the 10,821 singleton pregnancies (16.9 per 1000; 14.6–19.5). Outcome was known for 7 of the 14 anomalous multiple pregnancies (5 live births, 2 fetal losses).

The distribution by organ system is shown in Table 1 and Figure 1. Cardiac defects were the most frequent (81 pregnancies; 7.2 per 1000), followed by abdominal-wall defects (74; 6.6 per 1000), central nervous system anomalies (27; 2.4 per 1000), facial (23) and skeletal (23) anomalies (2.0 per 1000 each), and thoracic and urogenital anomalies (10 each; 0.9 per 1000). A cardiac and/or an abdominal-wall defect was present in 145 pregnancies (74% of the 197), including 10 with both. The individual entities are listed in Table 2. Atrioventricular septal defect (34) and hypoplastic left heart (18) accounted for two thirds of cardiac diagnoses; exomphalos (67; 6.0 per 1000) dominated the abdominal-wall group; alobar holoprosencephaly (10), open spina bifida (10), acrania/exencephaly (4) and encephalocele (4) made up the CNS group; and facial cleft (15), micrognathia (7) and limb reduction or positional defects (19) were the main facial and skeletal diagnoses. Congenital diaphragmatic hernia was diagnosed in 6 pregnancies and megacystis in 8.

The annual prevalence of first-trimester structural diagnoses was 49 per 1000 in 2011, when the centre received a high proportion of referred cases, and stabilised at 9–23 per 1000 from 2013 onwards without a clear temporal trend (Figure 2). No structural anomaly was recorded among the 136 pregnancies of the start-up period 2009–2010, and 3 were recorded among the 296 pregnancies of January–April 2026. In the sensitivity analyses, the prevalence was 16.1 per 1000 (95% CI 13.8–18.7; 163/10,141) after exclusion of 2009–2012, 18.0 per 1000 (15.6–20.7; 194/10,797) in the complete years 2011–2025, 19.0 per 1000 (16.5–21.8; 190/10,015) when one pregnancy per woman was analysed, and 16.9 per 1000 (14.6–19.5; 183/10,821) in singleton pregnancies.

**Table 1:** Structural anomalies identified at the 11–13^+6^-week scan by organ system (*n* = 11,229 pregnancies). A pregnancy is counted once per system and once in the total row; pregnancies with anomalies in several systems appear in each relevant row, so that system rows do not sum to the total.

System	Pregn.	Per 1000 (95% CI)	NT Median (mm)	NT ≥ 3.5 mm, *n* (%; 95% CI)	Abnormal Karyotype/Tested	TOP	Live Birth	Fetal/Neonatal Loss	Outcome Unknown
CNS	27	2.4 (1.7–3.5)	1.9	5 (18%; 8–37)	2/4	10	3	1	13
Face	23	2.0 (1.4–3.1)	2.7	10 (44%; 26–63)	2/5	6	5	0	12
Abdominal wall	74	6.6 (5.3–8.3)	2.0	19 (26%; 17–37)	5/14	12	18	2	42
Urogenital	10	0.9 (0.5–1.6)	2.2	1 (10%; 2–40)	0/1	2	2	1	5
Thorax	10	0.9 (0.5–1.6)	7.3	7 (70%; 40–89)	1/3	2	2	0	6
Heart	81	7.2 (5.8–9.0)	3.7	41 (51%; 40–61)	12/22	24	4	5	48
Abdomen	2	0.2 (0.0–0.6)	1.7	0 (0%; 0–66)	0/1	1	1	0	0
Skeletal	23	2.0 (1.4–3.1)	3.0	11 (48%; 29–67)	3/6	10	1	0	12
**Any structural anomaly**	**197**	**17.5 (15.3–20.1)**	**2.3**	**59 (30%; 24–37)**	**15/37**	**46**	**33**	**8**	**110**

NT, nuchal translucency; TOP, termination of pregnancy; CNS, central nervous system. Percentages with NT ≥ 3.5 mm are given with Wilson 95% confidence intervals; denominators for the thoracic, urogenital and abdominal groups are small (≤10) and the corresponding estimates are imprecise.

**Table 2:** Individual anomalies and markers recorded at the 11–13^+6^-week scan, with the first-trimester detectability category of Syngelaki et al. [5] (A, always detectable; B, sometimes detectable; C, usually not detectable in the first trimester). Pregnancies with several anomalies appear in more than one row; within a system, entity counts may therefore exceed the number of pregnancies in Table 1 (e.g., 2 pregnancies had exomphalos together with a body-stalk anomaly; overall, 10 pregnancies had two or more entities within a single system).

System	Anomaly	Cat.	*n*	NT Median	NT ≥ 3.5	Abn. Karyotype/Tested	TOP	Live Birth	Loss	Unknown
CNS	Acrania/exencephaly/anencephaly	A	4	1.6	0	0/0	1	0	0	3
CNS	Alobar holoprosencephaly	A	10	2.3	4	1/3	4	1	0	5
CNS	Encephalocele	B	4	1.8	0	0/0	1	1	0	2
CNS	Spina bifida	B	10	2.0	0	0/0	3	2	0	5
CNS	Other skull/brain	B	5	1.8	1	1/1	1	0	1	3
Face	Facial cleft	B	15	2.8	7	2/3	3	2	0	10
Face	Micrognathia	B	7	3.7	4	0/0	2	3	0	2
Face	Other facial (orbits, tumour, ears)	B	5	1.9	2	0/2	3	0	0	2
Abdominal wall	Exomphalos	A	67	1.9	19	5/13	11	17	2	37
Abdominal wall	Gastroschisis	A	6	2.0	0	0/1	1	1	0	4
Abdominal wall	Body stalk/other wall	A	3	1.3	0	0/0	2	0	0	1
Urogenital	Megacystis	A	8	2.0	1	0/1	2	0	1	5
Urogenital	Other renal/urinary	C	2	2.6	0	0/0	0	2	0	0
Thorax	Diaphragmatic hernia	B	6	4.0	3	0/1	0	2	0	4
Thorax	Other thoracic (effusion, lung)	B	4	8.6	4	1/2	2	0	0	2
Heart	Atrioventricular septal defect	B	34	3.6	17	7/11	13	2	3	16
Heart	Hypoplastic left heart	B	18	4.3	11	1/4	5	0	1	12
Heart	Hypoplastic right heart/univentricular	B	4	5.0	3	0/0	2	0	0	2
Heart	Ventricular septal defect	B	12	3.5	6	2/3	2	0	1	9
Heart	Tetralogy of Fallot	B	1	1.8	0	0/0	0	0	0	1
Heart	Transposition of great arteries	B	4	2.0	0	0/1	0	1	0	3
Heart	Coarctation of aorta	B	4	6.3	3	1/1	2	0	1	1
Heart	Other cardiac (Ebstein, DORV, RAA, valvular)	B	13	2.8	5	2/4	3	1	1	8
Abdomen	Ascites/abdominal cyst	B	2	1.7	0	0/1	1	1	0	0
Skeletal	Limb defects	B	19	4.0	11	3/6	9	0	0	10
Skeletal	Spine (kyphosis, scoliosis, other)	B	4	2.2	0	0/0	1	1	0	2
NT-related	Cystic hygroma	—	6	8.6	6	0/2	4	1	0	1
NT-related	Generalised oedema/hydrops	—	17	7.7	16	1/5	5	0	1	11
Marker	Nuchal oedema (marker)	—	37	5.3	37	2/10	9	2	2	24
Marker	Absent/hypoplastic nasal bone (marker)	—	19	3.3	9	3/3	6	3	0	10
Marker	ARSA/absent ductus venosus (marker)	—	13	2.8	6	1/1	3	1	0	9
Marker	Pericardial effusion (marker)	—	2	2.1	0	0/0	1	0	0	1

### 3.3. Nuchal Translucency, Combined Risk and Karyotype

NT was ≥3.5 mm in 59 of the 197 pregnancies with a structural anomaly (30%; 95% CI 24–37%) compared with 203 of the 11,032 pregnancies without (1.8%; 1.6–2.1%); OR 22.8 (95% CI 16.3–31.9), prevalence ratio 17.9 (13.5–23.7), *p* < 0.001, with cluster-robust standard errors by woman. The association was unchanged when one pregnancy per woman was analysed (OR 21.4, 95% CI 15.2–30.1). Conversely, of the 262 pregnancies with NT ≥ 3.5 mm, 59 (22.5%) had a structural anomaly identified at the same scan. The association with increased NT varied by system (Figure 3): NT ≥ 3.5 mm was present in 51% of cardiac defects, 48% of skeletal, 70% of thoracic and 43% of facial anomalies, but in only 26% of abdominal-wall, 19% of CNS and 10% of urogenital anomalies. The adjusted combined risk for trisomy 21 was ≥1:100 in 77 of 190 anomalous pregnancies with a risk estimate (40.5%); 28 anomalous fetuses had a high combined risk despite NT < 3.5 mm. A structural anomaly was present in 77 of 339 pregnancies with a high combined risk (22.7%) and in 113 of 10,739 with a low risk (1.1%; OR 27.6, 95% CI 20.2–37.9).

An invasive test result was recorded in the database for 37 of the 197 anomalous pregnancies (19%); a chromosomal abnormality was found in 15 (40.5% of those tested): trisomy 21 in 6, trisomy 18 in 6, triploidy in 2 and monosomy X in 1. Ten of the 15 chromosomally abnormal fetuses had NT ≥ 3.5 mm. Atrioventricular septal defect was the anomaly most often associated with aneuploidy (7 of 11 tested: trisomy 21 in 4 and trisomy 18 in 3), followed by exomphalos (5 of 13 tested).

### 3.4. Pregnancy Outcome

Outcome was known for 87 of the 197 anomalous pregnancies (44%): 46 were terminated (53% of those with known outcome), 33 ended in a live birth (38%), 7 in miscarriage or intrauterine death and 1 in a neonatal death. Termination was the predominant outcome for CNS (10 of 14 known), cardiac (24 of 33), skeletal (10 of 11) and facial anomalies (6 of 11), whereas 17 of the 30 exomphalos cases with known outcome resulted in a live birth, consistent with the high proportion of isolated and small exomphalos with normal karyotype. In the subgroup of 3105 pregnancies with known outcome, the prevalence of first-trimester structural diagnoses was 28.0 per 1000 (95% CI 22.8–34.4), higher than in the whole cohort, indicating that anomalous pregnancies were more likely to have their outcome recorded.

Anomalous pregnancies with known (*n* = 87) and unknown (*n* = 110) outcome are compared in Table S2. The two groups did not differ in maternal age, NT (median 2.5 vs. 2.2 mm; NT ≥ 3.5 mm in 32% vs. 28%), combined risk, anomaly category, organ system, multisystem involvement or documented invasive testing (all *p* > 0.2). The only difference was the year of screening: 62% of pregnancies with known outcome versus 31% of those with unknown outcome were screened in 2009–2017 (*p* < 0.001), reflecting the decline in outcome ascertainment over time. Missingness of outcome was therefore related to calendar period rather than to the type or severity of the anomaly. Of the 197 anomalous pregnancies, only 58 (29%) had a second-trimester examination at 18–24 weeks in the centre, compared with 74% of the whole cohort, consistent with termination or transfer of care after the first-trimester diagnosis.

**Figure 1:**
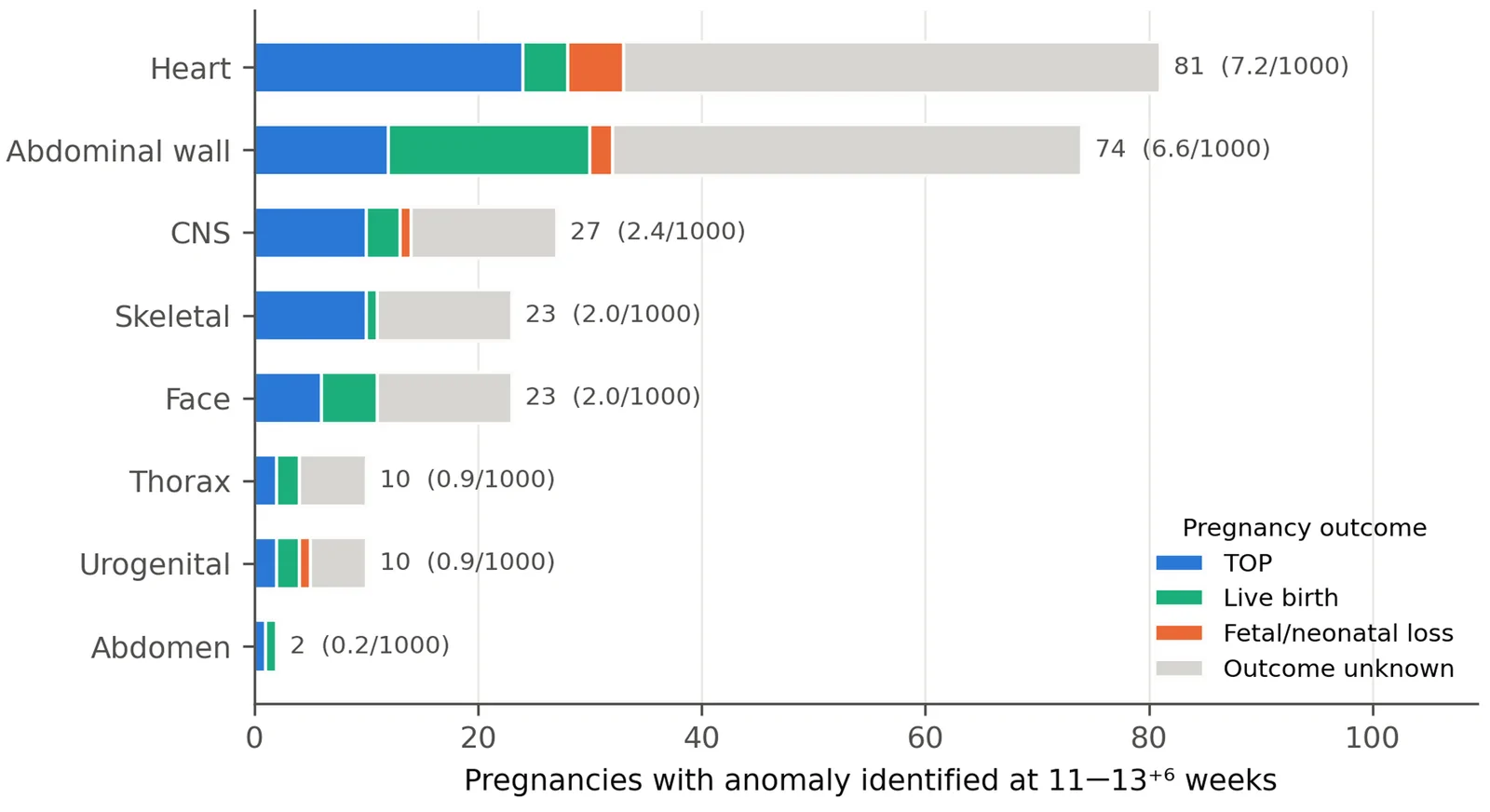
Pregnancies with a structural anomaly identified at 11–13^+6^ weeks by organ system, with pregnancy outcome. Labels give the number of pregnancies and the prevalence per 1000.

**Figure 2:**
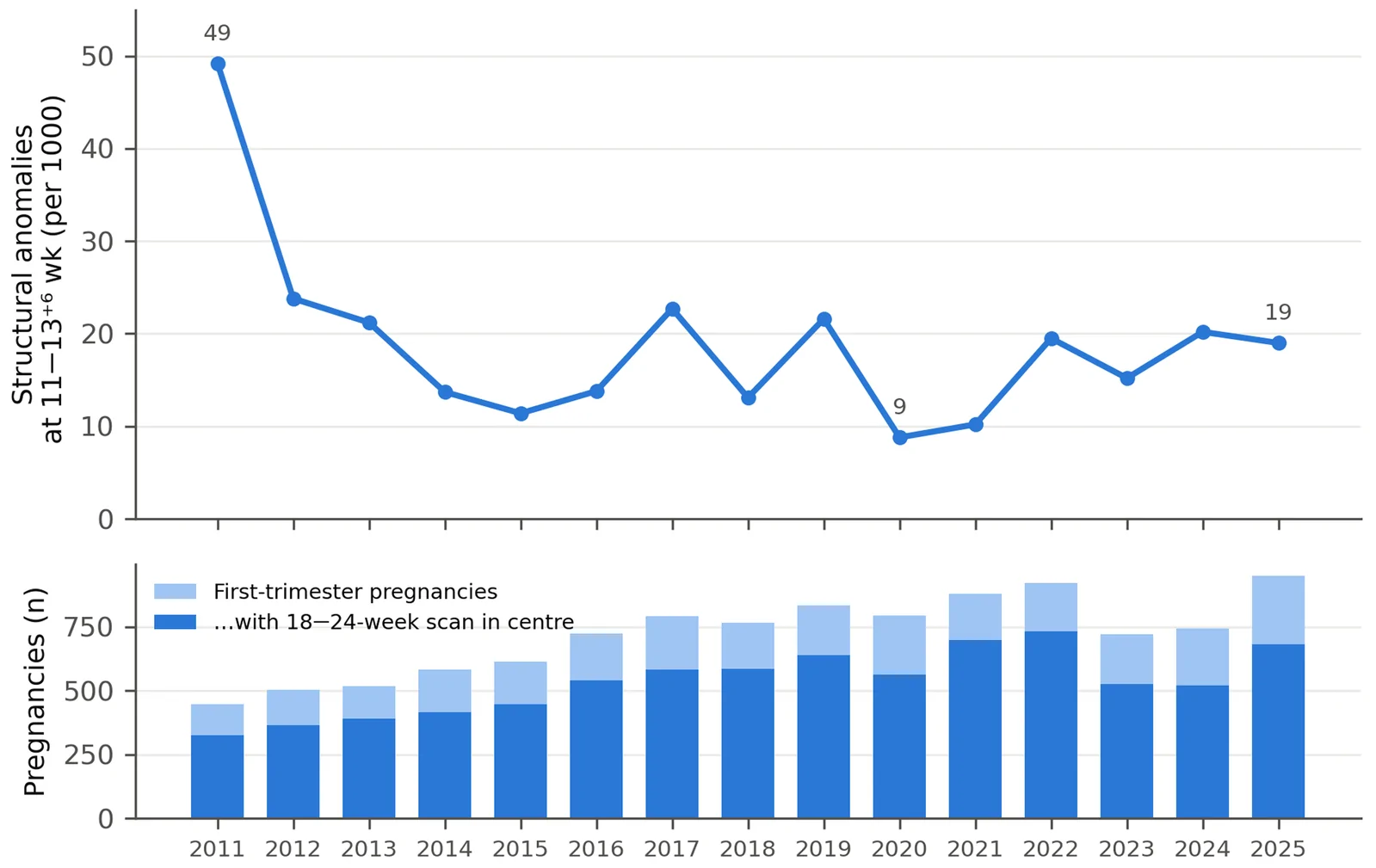
Annual prevalence of structural anomalies identified at the first-trimester scan (**top**) and annual number of first-trimester pregnancies with the proportion re-examined in the centre at 18–24 weeks (**bottom**), complete calendar years 2011–2025. The start-up period August 2009–December 2010 (136 pregnancies, no structural anomaly) and the partial year January–April 2026 (296 pregnancies, 3 anomalies) are not plotted and are given in Table S1.

**Figure 3:**
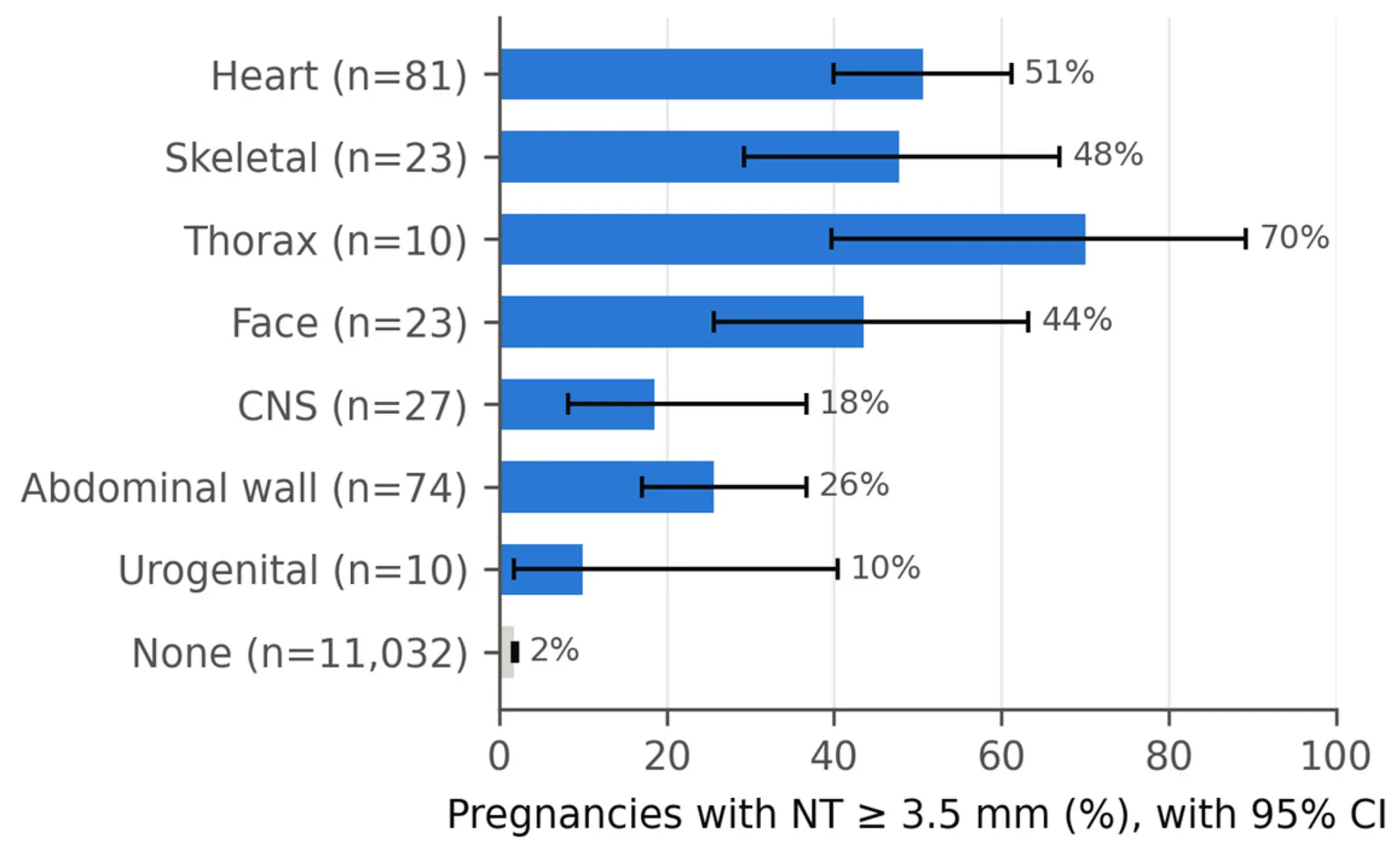
Proportion of pregnancies with NT ≥ 3.5 mm, with Wilson 95% confidence intervals, according to the organ system involved among pregnancies with a first-trimester structural anomaly; pregnancies without a structural anomaly are shown as the reference group (grey). Denominators are given in parentheses; estimates for systems with ≤ 10 pregnancies are imprecise.

## 4. Discussion

### 4.1. Main Findings

In this cohort of more than 11,000 pregnancies undergoing combined screening in a single Romanian fetal medicine centre, a screening population with referral enrichment, a structural anomaly was identified at the 11–13^+6^-week scan in 1.75% of pregnancies (1.6% after exclusion of 2009–2012). The spectrum was dominated by cardiac and abdominal-wall defects, which together were present in 74% of the anomalous pregnancies, followed by central nervous system, facial and skeletal anomalies. Almost one in five anomalous fetuses had multisystem involvement, one third had an NT of 3.5 mm or more, and 41% of those with a documented invasive test had a chromosomal abnormality. Where outcome was known, half of the anomalous pregnancies were terminated and 38% resulted in a live birth, with a markedly better prognosis for exomphalos than for cardiac, CNS or skeletal anomalies. Increased NT was strongly associated with the presence of a structural anomaly (OR 22.8), and the association was robust to clustering of pregnancies within women.

### 4.2. Comparison with the Literature

The overall prevalence of first-trimester structural diagnoses in our cohort (17.5 per 1000) is higher than the 4–5 per 1000 that can be derived from the Fetal Medicine Foundation series, in which 27.6% of 1720 major non-chromosomal abnormalities among 100,997 singleton pregnancies were diagnosed at 11–13 weeks [5], and higher than most figures that can be derived from other routine screening programmes [11,12]. A comparison with published series is shown in Table 3. When first-trimester detection rates are converted into the number of anomalies identified at 11–13 weeks per 1000 screened pregnancies, the published figures range from about 2 per 1000 in nationwide programmes with restricted anomaly lists [8] to 5 per 1000 in the FMF series [5] and 13 per 1000 in a Chinese single-centre programme with a broad definition [12]; a recent Turkish tertiary series reported abnormal first-trimester findings in 1.69% of pregnancies [13], almost identical to the 1.75% observed here. Several factors are likely to contribute to the position of our cohort at the upper end of this range. First, the most likely explanation for the relatively high frequency of first-trimester structural diagnoses is the referral function of the centre. Women with suspected abnormalities identified elsewhere were referred for specialist assessment, but this indication was not coded as a structured field and such pregnancies cannot be separated retrospectively from routine screening attendances. Referral enrichment was particularly apparent in the early years of the series, most evidently in 2011, when the prevalence was 49 per 1000. Exclusion of 2009–2012 lowered the estimate from 17.5 to 16.1 per 1000, supporting some contribution from the early referral-enriched period, although this temporal sensitivity analysis cannot quantify the magnitude of referral bias. The referral component is also visible in the excess of exomphalos (6.0 per 1000 compared with approximately 2.5 per 1000 in the FMF series [14]), including small exomphalos that may resolve and that are frequently isolated with a normal karyotype [14]. Second, our definition included all entries in the anatomy checklist, including ventricular septal defects, isolated micrognathia and positional limb defects that may not qualify as major anomalies in registry definitions. Third, chromosomally abnormal fetuses were included, whereas the FMF analyses were restricted to euploid fetuses. The distribution across organ systems, however, closely resembles the published pattern: cardiac defects, abdominal-wall defects and CNS anomalies are the three leading groups in the first-trimester series of Syngelaki et al. [4,5], Iliescu et al. [9] and Karim et al. [6], and the relative rarity of renal, gastrointestinal and pulmonary diagnoses in the first trimester is consistent with the natural history of these conditions, most of which are not detectable before 18–22 weeks. The same gradient is visible in anomaly-specific detection rates: in the FMF series all cases of acrania, alobar holoprosencephaly, exomphalos, gastroschisis and body-stalk anomaly, 92.5% of hypoplastic left heart, 90.9% of atrioventricular septal defect and 59% of open spina bifida were identified at 11–13 weeks, whereas ventriculomegaly, isolated cleft lip, ventricular septal defect, hydronephrosis and talipes were rarely identified [5]; Liao et al. reported first-trimester detection of 95.6% for abdominal-wall defects, 66.3% for central nervous system, 57.9% for major cardiac, 33.8% for limb and 21.2% for urogenital anomalies [12]. The composition of our first-trimester series, with exomphalos, atrioventricular septal defect, hypoplastic left heart and holoprosencephaly as the leading entities and very few renal or gastrointestinal diagnoses, is therefore what would be expected from a screening population examined with a standard protocol. A further potential source of temporal variation is the evolution of the recommended first-trimester anatomical survey between the 2013 ISUOG guideline [2] and its 2023 update [3], which expanded the cardiac and posterior-brain views. In our centre the ISUOG recommendations current at the time served as the reference for the anatomical survey, without a separate written local protocol, and the structured checklist of the reporting database was unchanged; a gradual broadening of the survey, together with improvements in equipment and operator experience, may therefore have contributed to temporal variation in detection. However, the prevalence of first-trimester diagnoses was highest in the earliest years of the series, before the extended survey was recommended, which argues against an expansion of the protocol as the explanation for the early peak and is more consistent with referral enrichment. A change in recording practice also contributed to the early excess of exomphalos. In the early years, small or possibly transient exomphalos identified at a small crown–rump length were recorded as abnormal findings at the initial examination and the pregnancy was subsequently re-examined. With increasing experience, because many of these early findings were not confirmed at follow-up, the centre adopted a practice of recording exomphalos as present only once the CRL had reached 55 mm. This change is consistent with the excess of exomphalos in 2011 (10 of 22 structural findings, most resulting in live births) and should be considered when interpreting temporal variation in the series.

The strong association between structural anomalies and increased NT confirms the findings of Souka et al. [16] and of the FMF studies, which reported major anomalies in 10–25% of euploid fetuses with NT ≥ 3.5 mm, with cardiac defects being the most common. In the FMF series of 93,209 pregnancies, 36.5% of fetuses with a major heart defect had increased NT and 53.6% of major heart defects were diagnosed at the first-trimester scan [15]. This corresponds to 1.2 major heart defects identified at 11–13 weeks per 1000 pregnancies, compared with 7.2 per 1000 cardiac diagnoses in our cohort, a difference that reflects our inclusion of ventricular septal defects and of chromosomally abnormal fetuses as well as referral of suspected cases. In our cohort 22.5% of pregnancies with NT ≥ 3.5 mm had a structural anomaly at the same scan, and half of all cardiac defects presented with increased NT, supporting the recommendation for a detailed cardiac assessment at 11–13 weeks and again at 16 and 20 weeks in fetuses with increased NT [3,17]. The finding that 40% of anomalous fetuses had a high combined risk, and that 28 had a high risk with NT below 3.5 mm, illustrates that the risk algorithm and the anatomical survey identify partly overlapping populations. The high rate of aneuploidy among tested fetuses, particularly in atrioventricular septal defect and exomphalos, mirrors the classical associations with trisomy 21 and 18 [4,14] and argues for offering diagnostic testing with chromosomal microarray whenever a structural anomaly is seen in the first trimester, irrespective of the combined risk [3].

Data from Central and Eastern Europe are limited. Iliescu et al. reported a first-trimester detection rate of 76% for major anomalies using an extended protocol in a Romanian tertiary population of 5472 pregnancies [9], and Tudorache et al. showed that a detailed cardiac examination is feasible at 12–13 weeks in the same setting [10]. Our cohort complements these studies by describing a large screening population with referral enrichment, with a longer observation period and a wider range of anomalies, and by quantifying the relationship between first-trimester diagnoses and NT and karyotype in this population.

### 4.3. Clinical Implications

Early identification of structural anomalies during first-trimester screening has several clinical implications. It enables diagnostic genetic testing to be offered early in pregnancy and allows timely referral for detailed fetal assessment and multidisciplinary counselling. For continuing pregnancies, early diagnosis also provides additional time for serial evaluation and appropriate planning of subsequent prenatal care and delivery. Conversely, the predominance of termination among cardiac, central nervous system and skeletal anomalies in our cohort reflects the severity of the abnormalities detectable at this gestation and underscores the importance of accurate diagnosis and non-directive counselling. The substantial proportion of pregnancies whose outcome was not returned to the screening centre also illustrates the difficulty of longitudinal ascertainment when prenatal screening and delivery take place in different institutions. More broadly, the 11–13-week visit has become the focal point of an inverted pyramid of prenatal care in which anatomical assessment, aneuploidy screening and the prediction of obstetric complications are combined [18]; the early identification of major structural anomalies, illustrated by case reports of diagnoses made as early as the first-trimester scan [19], allows counselling and pregnancy management to begin at a stage when the full range of options remains available.

### 4.4. Strengths and Limitations

The strengths of the study are the size of the cohort, the screening setting, the prospective and structured recording of anatomy in a single database by a small group of operators over more than 16 years, and the availability of NT, combined risk and karyotype for the same pregnancies. The main limitation is the incompleteness of outcome ascertainment, which was available for 28% of all pregnancies and 44% of anomalous pregnancies and declined over time as the proportion of women delivering outside the centre increased. Anomalous pregnancies were more likely to have a recorded outcome, and those with known and unknown outcome had similar anomaly types, NT, combined risk and maternal age, differing only in the calendar period of screening (Table S2); the outcome distribution reported here is therefore unlikely to be biased by anomaly severity, although residual bias related to place of delivery cannot be excluded. However, the absence of postnatal information for the majority of pregnancies means that false-negative first-trimester scans could not be identified; the prevalence figures therefore refer to prenatally identified anomalies and should not be read as detection rates. Invasive testing results were recorded for only one fifth of anomalous pregnancies, which precludes an estimate of the true aneuploidy rate. Confirmation of the prenatal diagnosis by autopsy or postnatal examination was not systematically available, and some first-trimester diagnoses, in particular small exomphalos, ventricular septal defects and isolated micrognathia, may have been transient or revised at a later scan; false-positive first-trimester cardiac diagnoses have been reported in about one in eight confirmed cases even in experienced hands [20], and the proportion of such findings in our series could not be quantified. Finally, although the centre provides routine combined screening, a proportion of women attended after an abnormal finding elsewhere, particularly in the early years of the series; the prevalence of 1.75% therefore reflects a screening population with referral enrichment and should not be regarded as a population-based prevalence. Although second-trimester follow-up was available for approximately three quarters of the cohort, the historical second-trimester extract contained organ-system abnormality flags rather than sufficiently granular anomaly-specific fields. Because these flags also capture minor findings and soft markers, anomalies first identified after a normal first-trimester scan could not be classified reliably in the present study. Anomaly-specific linkage and adjudication of the longitudinal examinations are being undertaken separately and will allow prenatal detection rates to be estimated in this population.

## 5. Conclusions

In a large single-centre screening population with referral enrichment from Romania, a structural anomaly was identified at the 11–13^+6^-week scan in 1.75% of pregnancies (1.6% after exclusion of 2009–2012), with cardiac and abdominal-wall defects accounting for most cases. These figures describe prenatally identified anomalies in a fetal medicine centre and should not be read as population-based prevalence or as detection rates. The strong association of these diagnoses with increased NT and chromosomal abnormalities supports the integration of systematic anatomical assessment and genetic counselling into first-trimester screening.

## Figures and Tables

**Table 3:** Published series of first-trimester ultrasound detection of structural anomalies compared with the present cohort. Detection rate (DR) is the proportion of all prenatally and postnatally diagnosed anomalies identified at the first-trimester scan; the last column (anomalies identified at 11–13^+6^ weeks per 1000 screened pregnancies) is derived as prevalence × DR where both are reported and is the only measure directly comparable with the present study. † Restricted list of major anomalies (ACCEPTS target conditions). ‡ Pregnancies with abnormal first-trimester findings (55/3254 = 1.69%).

Study	Setting	Pregnancies (*n*)	Anomalies, *n* (%)	First-Trimester DR	Identified at T1 per 1000 Pregnancies
Syngelaki et al., 2019 [5]	UK, FMF (routine screening, singletons)	100,997	1720 (1.7%)	27.6%	4.7
Karim et al., 2017 [6] (meta-analysis)	19 studies, low-risk/unselected	115,731	0.93–1.63%	46.1% (major); 32.4% (all)	≈4–7
Karim et al., 2025 [8]	England, nationwide, 110 units	1,030,224	5895 (0.57%) †	32.7% (<16 wk)	1.9
Liao et al., 2021 [12]	China, single centre, routine protocol	53,349	1578 (3.0%)	43.1%	12.7
Iliescu et al., 2013 [9]	Romania, tertiary, extended protocol	5472	—	76.3% (major structural defects); 40.6% (all abnormalities)	—
Minnella et al., 2020 [15] (heart only)	UK, FMF, routine screening	93,209	211 major heart defects (2.3‰)	53.6%	1.2 (cardiac)
Dilek et al., 2026 [13]	Turkey, two tertiary units	3254	—	60.4%	16.9 ‡
Present study	Romania, single screening centre	11,229	—	not estimable	17.5 (7.2 cardiac)

## Data Availability

The pseudonymised dataset is held by the Maternal-Fetal Medicine Center, Timișoara, and is not publicly available because of patient-confidentiality restrictions; aggregate data supporting the findings are available from the corresponding author on reasonable request.

## Supplementary Materials

The following supporting information can be downloaded at https://www.mdpi.com/article/10.3390/medicina62101824/s1, Table S1: Annual number of first-trimester pregnancies, structural diagnoses, second-trimester attendance and outcome ascertainment, 2009–2026; Table S2: Characteristics of anomalous pregnancies with known versus unknown pregnancy outcome.
