# Supplementary material for: Structural Fetal Anomalies Identified During First-Trimester Screening: A Single-Centre Experience in Romania
Source: Medicina (Kaunas). 2026 Sep 22;62(10):1824. doi: 10.3390/medicina62101824 (PMC13707282; doi:10.3390/medicina62101824)
Supplement: Supplementary file 1 [file medicina-62-01824-s001.zip › medicina-4583733-supplementary.pdf]

Supplementary Materials

Structural Fetal Anomalies Identified During First-Trimester Screening: A Single-Centre Experience in Romania

Table S1. Annual number of first-trimester pregnancies, structural anomalies identified at the 11–13^{+6}-week scan, second-trimester attendance (18–24 weeks) in the centre and outcome ascertainment, August 2009–April 2026 (2009: from August; 2026: January–April).

Year of first-trimester scan	Pregnancies (n)	Structural anomaly at 11–13^{+6} wk (n)	Per 1000	18–24-week scan in centre (n)	Outcome known (n)
2009	50	0	0.0	0	3
2010	86	0	0.0	48	59
2011	447	22	49.2	325	261
2012	505	12	23.8	364	297
2013	518	11	21.2	392	277
2014	583	8	13.7	415	325
2015	615	7	11.4	447	281
2016	725	10	13.8	541	236
2017	793	18	22.7	582	328
2018	765	10	13.1	585	161
2019	833	18	21.6	638	149
2020	796	7	8.8	564	120
2021	880	9	10.2	700	110
2022	923	18	19.5	733	170
2023	722	11	15.2	527	146
2024	743	15	20.2	522	94
2025	949	18	19.0	681	82
2026	296	3	10.1	221	6

Table S2. Characteristics of pregnancies with a structural anomaly identified at the 11–13^{+6}-week scan, according to whether pregnancy outcome was known (n = 197). Values are median (IQR) or n (%). p-values from the Mann–Whitney U test (continuous) or Fisher's exact test (categorical).

Characteristic	Outcome known (n=87)	Outcome unknown (n=110)	p
Maternal age, years	30.8 (27.1–35.2)	30.9 (27.5–35.9)	0.44
Maternal age ≥ 35 years	23 (26%)	34 (31%)	0.53
Year of screening	2016 (2012–2022)	2020 (2017–2023)	< 0.001
Screened 2009–2017	54 (62%)	34 (31%)	< 0.001
NT, mm	2.5 (1.7–3.9)	2.2 (1.6–4.3)	0.683
NT ≥ 3.5 mm	28 (32%)	31 (28%)	0.639
Combined risk ≥ 1:100	35 (40%)	42 (38%)	0.771
Category A anomaly	38 (44%)	53 (48%)	0.567

Characteristic	Outcome known (n=87)	Outcome unknown (n=110)	p
Cardiac anomaly	33 (38%)	48 (44%)	0.467
Abdominal-wall anomaly	32 (37%)	42 (38%)	0.883
Multisystem involvement	16 (18%)	21 (19%)	1.0
Invasive test recorded	20 (23%)	17 (15%)	0.201
Chromosomal abnormality recorded	8 (9%)	7 (6%)	0.59

NT, nuchal translucency. Category A: anomalies always detectable at 11–13 weeks (Syngelaki et al. 2019). Combined risk: adjusted first-trimester combined risk for trisomy 21. Invasive test recorded: result of chorionic villus sampling or amniocentesis entered in the database.
